# New insights into fibrous cap thickness of vulnerable plaques assessed by optical coherence tomography

**DOI:** 10.1186/s12872-022-02896-z

**Published:** 2022-11-12

**Authors:** Xianglan Liu, Wujian He, Xulin Hong, Duanbin Li, Zhezhe Chen, Yao Wang, Zhaoyang Chen, Yi Luan, Wenbin Zhang

**Affiliations:** 1 Department of Cardiology, Sir Run Run Shaw Hospital, Zhejiang University School of Medicine, No. 3 Qingchun Dong Road, Jianggan District, Hangzhou, China; 2 Key Laboratory of Cardiovascular Intervention and Regenerative Medicine of Zhejiang Province, Hangzhou, China; 3 Cardiology Department, Union Hospital, Fujian Medical University, 29 Xin-Quan Road, Fuzhou, 350001 Fujian China

**Keywords:** Vulnerable plaque, Fibrous cap thickness, Optical coherence tomography, Plaque rupture, Thrombus

## Abstract

**Objective:**

Vulnerable plaques with fibrous cap thickness (FCT) of ≤65 μm are prone to rupture and/or thrombosis. However, plaques with FCT > 65 μm cause acute myocardial infarction and even sudden death. We aimed to investigate the relationship between 65 < FCT ≤ 80 μm and plaque rupture and/or thrombosis using optical coherence tomography (OCT).

**Methods:**

OCT was performed on culprit lesions in 502 consecutively enrolled patients to identify FCT. Patients were classified into three groups according to FCT: Group A (FCT ≤ 65 μm, *n* = 147), Group B (65 < FCT ≤ 80 μm, *n* = 84) and Group C (FCT > 80 μm, *n* = 271). Clinical and laboratory data was collected from the inpatient medical record system.

**Results:**

Plaques with thinner FCT, especially < 65 μm, were more susceptible to rupture and/or thrombosis (*P* < 0.001). Plaques with FCT between 65 and 80 μm had a higher probability of rupture and/or thrombosis than those with FCT > 80 μm (*P* < 0.001). In multivariable analysis, FCT ≤ 65 μm and 65 < FCT ≤ 80 μm were independent predictors for plaque rupture ([FCT ≤ 65 μm vs. FCT > 80 μm]: OR = 8.082, 95% CI = 4.861 to 13.435, *P* < 0.001; [65 < FCT ≤ 80 μm vs. FCT > 80 μm]: OR = 2.463, 95% CI = 1.370 to 4.430, *P* = 0.003), thrombosis ([FCT ≤ 65 μm vs. FCT > 80 μm]: OR = 25.224, 95% CI = 13.768 to 46.212, *P* < 0.001; [65 < FCT ≤ 80 μm vs. FCT > 80 μm]: OR = 3.675, 95% CI = 2.065 to 6.542, *P* < 0.001) and plaque rupture with thrombosis ([FCT ≤ 65 μm vs. FCT > 80 μm]: OR = 22.593, 95% CI = 11.426 to 44.674, *P* < 0.001; [65 < FCT ≤ 80 μm vs. FCT > 80 μm]: OR = 4.143, 95% CI = 1.869 to 9.184, *P* < 0.001).

**Conclusions:**

OCT-assessed 65 < FCT ≤ 80 μm was independently associated with increased risk of plaque rupture and/or thrombosis compared with FCT > 80 μm.

**Supplementary Information:**

The online version contains supplementary material available at 10.1186/s12872-022-02896-z.

## Introduction

Coronary plaque rupture frequently leads to acute coronary syndrome (ACS), which is a major cause of morbidity and mortality in patients with coronary artery disease (CAD). Early detection of plaques that prone to rupture, also known as vulnerable plaques, is one of the important advances in ACS prevention. Vulnerable plaques are commonly characterized by large lipid cores, thin fibrous caps, and macrophage infiltration. A thin-cap fibroatheroma (TCFA) is currently defined as a plaque with a fibrous cap < 65 μm overlying a large lipid pool [1]. However, evidence showed that plaques with fibrous caps > 65 μm are also susceptible to plaque rupture or erosion and can cause acute myocardial infarction (AMI) or sudden death [2]. One possible reason is that this threshold was obtained via autopsy analysis of plaques that were already ruptured, which may be inconsistent and under-estimated with the situation in vivo.

High-resolution OCT (10–20 μm) has emerged as one of the most promising intravascular imaging modalities for microstructure evaluation of plaques, including fibrous cap thickness (FCT), lipid core arc, macrophage accumulation, cholesterol crystals, calcifications, thrombi and micro vessels [3]. Yonetsu et al. [2] showed that nearly all ruptured plaques have a FCT < 80 μm measured by OCT. Furthermore, Brown et al. [4] demonstrated that FCT<85 μm might be the optimal threshold of identifying histopathologic TCFAs. Current OCT-based findings are inconsistent with those of a previous autopsy study. Therefore, in this study, we further investigated the correlation between 65 μm < FCT ≤ 80 μm and plaque rupture or thrombosis using OCT.

## Materials and methods

### Study population

From January 2013 to December 2018, we recruited a retrospective cohort of 502 patients aged 18 to 85 who underwent OCT imaging before percutaneous coronary intervention (PCI) of identifiable new single culprit lesions in their primary coronary arteries. Of these patients, 245 had stable angina pectoris and 257 had ACS (unstable angina pectoris and acute myocardial infarction). The exclusion criteria were as follows: (1) lesions with thrombolysis in myocardial infarction (TIMI) flow ≤2 after thrombectomy; (2) lesions in a bypass graft; (3) chronic total occluded coronary arteries; (4) inability to insert the imaging catheter with or without predilation; and (5) patients with severe valve diseases, advanced heart failure, previous MI, a history of any other revascularization procedures or strokes. Information of patients was collected, including risk factors, medication usage, family history and laboratory results. Before coronary angiography, all patients were prescribed aspirin and clopidogrel/ticagrelor as part of the standard treatment process. Study protocol complied with the Declaration of Helsinki and was approved by the Research Ethics Committee of the Sir Run Run Shaw Hospital, Zhejiang University School of Medicine, China. All patients signed an informed consent form upon recruiting.

### Quantitative coronary angiography analysis

Patients were pretreated with 300 mg of aspirin, 300 mg of clopidogrel or 180 mg of ticagrelor, and 100 U/kg of heparin. Coronary angiography was performed via a transradial or transfemoral approach with the use of a 6F or 7F sheath after intracoronary administration of 100–200 mg nitroglycerine. Angiographic images were analysed using a quantitative coronary angiogram analysis program (CAAS 5.10.1, Pie Medical Imaging BV, Maastricht, Netherlands) individually by two experienced technicians blinded to clinical features of patients. The parameters of quantitative coronary angiography (QCA) included lesion length, reference vessel diameter (RVD), minimal lumen diameter (MLD) and diameter stenosis (DS). RVD was defined as the average diameter of the distal and proximal reference diameters. DS was calculated as follows: DS = (RVD - MLD)/RVD × 100%.

### OCT data acquisition and analysis

OCT was performed with a commercially available frequency-domain OCT C7XR system and a Dragonfly catheter (LightLab Imaging/St. Jude Medical) as previously reported [5]. OCT images were automatically recorded then analysed using a previously reported approach by two experienced technicians blinded to the clinical information; these technicians jointly evaluated the images with proprietary offline software (LightLab Imaging).

OCT-based parameters, including FCT, lipid arc, lipid length, ruptured plaques, thrombus, microchannels, cholesterol crystals, calcium node and macrophages, were evaluated. FCT measurements were performed individually at the thinnest part of each lesion for three times by two technicians, then the mean value was calculated; the minimum FCT value in each plaque was selected. The lipid arc was measured at 1-mm intervals throughout the entire lesion. A microvessel was defined as a sharply delineated signal-poor void with a diameter of 50–300 μm that was not connected to the vessel lumen and was noted on more than three consecutive frames. Calcification was characterized as a heterogeneous area with low signal intensity and sharp borders. A thrombus was defined as a mass floating in or protruding into the lumen with a diameter of at least 250 μm [22421299].

### Statistical analysis

All statistical analyses were performed using SPSS analytical software (version 22.0; SPSS, Inc., Chicago, Illinois, USA) and R version 3.5.1 (The R Foundation for Statistical Computing, Vienna, Austria). Categorical variables were presented as counts (proportions) and compared using the chi-square test or Fisher’s exact test. Continuous variables were presented as the means and standard deviations or the medians and interquartile ranges, based on their distribution. The independent two-sample 𝑡-test or one-way analysis of variance (ANOVA) with post hoc least significance difference (LSD) test was used to assess the differences between multiple sets of data. Univariable and multivariable logistic regression analyses were used to assess the relationship between FCT and plaque rupture and/or thrombosis. Receiver operating characteristic (ROC) analysis was used to determine the best cut-off cap thickness values for discriminating between ruptured and non-ruptured plaques or thrombosis and non-thrombosis. We further used restricted cubic splines with five knots at the 10th, 30th, 50th, 70th, and 90th centiles to flexibly model the association of FCT with plaque rupture or thrombosis. A *p*-value of less than 0.05 was considered as statistically significant.

## Results

In our study, the optimal cap thickness values measured by OCT for predicting plaque rupture, thrombosis or plaque rupture with thrombosis were 81.5 μm, 78.5 μm, or 74.5 μm, respectively, by ROC analysis (Supplement Fig. 1). Patients were classified into three groups according to FCT: Group A (FCT ≤ 65 μm, *n* = 147), Group B (65 < FCT ≤ 80 μm, *n* = 84) and Group C (FCT > 80 μm, *n* = 271).

### Clinical characteristics and laboratory results

The clinical characteristics of the patients are summarized in Table 1. There were no statistically significant differences in terms of age, sex, or other important coronary risk factors among groups. Patients with thinner FCT were more prone to ACS (78.2, 56.0 and 35.1%, *P* < 0.001 for groups A, B and C, respectively). Patients with plaques of 65 < FCT ≤ 80 μm had a higher prevalence of ACS than those with plaques of FCT > 80 μm (56.0% vs. 35.1%, *P* < 0.001). No significant differences were found in the laboratory data between Group B and Group C in addition to blood creatinine level.

**Table 1:** Baseline characteristics (LSD)

	Group A FCT ≤ 65 μm (*n* = 147)	Group B 65 < FCT ≤ 80 μm (*n* = 84)	Group C FCT > 80 μm(*n* = 271)	*P*-value
Age, years	66.0 ± 11.5	65.4 ± 10.7	65.6 ± 10.3	0.921
Male, n (%)	127 (86.4)	70 (83.3)	220 (81.2)	0.397
Current smoker, n (%)	66 (44.9)	32 (38.1)	95 (35.1) △	0.142
Hypertension, n (%)	102 (69.4)	59 (70.2)	173 (63.8)	0.379
Hypercholesterolaemia, n (%)	81 (55.1)	45 (53.6)	157 (57.9)	0.729
Diabetes mellitus, n (%)	45 (30.6)	28 (33.3)	92 (33.9)	0.782
Haemodialysis, n (%)	3 (2.0)	1 (1.2)	9 (3.3)	0.591
Previous MI, n (%)	14 (9.5)	12 (14.3)	56 (20.7) △	**0.011**
Previous PCI, n (%)	19 (12.9)	19 (22.6)	73 (26.9) △	**0.004**
Previous CABG, n (%)	3 (2.0)	2 (2.4)	3 (1.1)	0.504
Indication for index PCI, n (%)				
SAP	32 (21.8)	37 (44.0) *	176 (64.9) #△	**< 0.001**
ACS	115 (78.2)	47 (56.0) *	95 (35.1) #△	**< 0.001**
Laboratory results				
TC, mg/dl	194.9 ± 42.5	185.9 ± 34.0	180.2 ± 38.8 △	**0.001**
LDL-C, mg/dl	123.0 ± 36.7	113.7 ± 27.5 *	107.6 ± 34.2 △	**< 0.001**
HDL-C, mg/dl	44.4 ± 11.4	44.3 ± 9.8	46.3 ± 11.6	0.172
Triglyceride, mg/dl	132.3 ± 84.0	131.2 ± 91.2	133.6 ± 71.2	0.968
Creatinine, mg/dl	0.99 ± 0.97	0.89 ± 0.58	1.12 ± 1.56 #	0.286
eGFR, ml/(min × 1.73 m^2^)	72.5 ± 23.6	71.9 ± 18.9	70.8 ± 23.8	0.770
hs-CRP, mg/L	0.50 ± 0.93	0.37 ± 0.61	0.35 ± 0.91	0.082
HbA1c, %	6.1 ± 1.4	5.9 ± 0.82	6.0 ± 1.2	0.628
Previous medications, n (%)				
Statins	10 (6.8)	12 (14.3)	52 (19.2) △	**0.003**

Data are n (%) or mean ± SD

*LDL-C* low-density lipoprotein cholesterol, *HDL-C* high-density lipoprotein cholesterol, *CK* creatine kinase, *hs-CRP* high-sensitivity C-reactive protein, *TC* total cholesterol

∗Significance between FCT ≤ 65 μm group and 65 < FCT ≤ 80 μm group; #significance between 65 < FCT ≤ 80 μm group and FCT > 80 μm group; △significance between FCT ≤ 65 μm group and FCT>80 μm group. *P*-value < 0.05 was in bold

### Angiographic findings

The angiographic data are presented in Table 2. We found that the right coronary artery (RCA) was the most frequent culprit vessel compared to the left anterior descending (LAD) artery and left circumflex artery (LCX). Lesions in RCA were seen more frequently in Group A, and those in LAD were more frequently seen in Group C. The MLD and DS were significantly different among three groups.

**Table 2:** Angiographic findings

	Group A FCT ≤ 65 μm (*n* = 147)	Group B 65 < FCT ≤ 80 μm (*n* = 84)	Group C FCT > 80 μm(*n* = 271)	*P*-value
Culprit vessel, n (%)				
Left anterior descending, n (%)	57 (38.8)	35 (41.7)	158 (58.3) #△	**< 0.001**
Left circumflex, n (%)	28 (19.0)	20 (23.8)	38 (14.0) #	0.088
Right coronary artery, n (%)	62 (42.2)	29 (34.5)	75 (27.7) △	**0.010**
QCA findings				
MLD, mm	0.96 ± 0.63	1.15 ± 0.91	1.26 ± 0.90 △	**0.002**
RLD, mm	7.35 ± 4.41	7.85 ± 3.07	7.07 ± 2.73	0.527
DS, %	84.5 ± 9.2	82.8 ± 13.7	79.4 ± 10.4 △	**0.038**
Lesion length, mm	24.6 ± 8.0	23.5 ± 7.9	23.6 ± 8.7	0.416

*MLD* minimal lumen diameter, mm, *RLD* reference lumen diameter, mm, *DS* diameter stenosis

#significance between 65 < FCT ≤ 80 μm group and FCT>80 μm group; △significance between FCT ≤ 65 μm group and FCT>80 μm group. Values are n(%)

### OCT findings

Culprit sites were successfully assessed in all subjects without any serious procedural complication. The OCT data is presented in Table 3. The vulnerable plaque lesions in Group A showed a larger lipid arc (165.4 ± 47.1 vs. 127.5 ± 42.9, *P* < 0.001) and longer lipid length (13.8 ± 6.2 vs. 9.7 ± 5.8, *P* < 0.001) than those in Group C. The prevalence rates of calcium nodes, macrophages, and cholesterol crystals were not significantly different among the three groups. Significant differences in macrophage length, macrophage volume index, plaque rupture and thrombus were observed among patients with different FCT. Plaques with a FCT ≤ 65 μm were more likely to end up with plaque rupture and thrombus than those with a 65 < FCT ≤ 80 μm and FCT > 80 μm. Plaques with a 65 < FCT ≤ 80 μm had larger lipid arcs (158.4 ± 45.5 vs. 127.5 ± 42.9, *P* < 0.001) and longer lipid lengths (13.5 ± 7.0 vs. 9.7 ± 5.8, *P* < 0.001), as well as longer macrophage lengths, larger macrophage volume indices and a higher probability of plaque rupture and thrombosis than plaques with a FCT > 80 μm, which indicates the vulnerability of this kind of plaques.

**Table 3:** Optical coherence tomography findings

	Group A FCT ≤ 65 μm (*n* = 147)	Group B 65 < FCT ≤ 80 μm (*n* = 84)	Group C FCT > 80 μm(*n* = 271)	*P* value
Thin-cap thickness. μm	56.7 ± 6.3	72.9 ± 5.3 *	142.6 ± 56.0 #△	**< 0.001**
Mean lipid arc, °	165.4 ± 47.1	158.4 ± 45.5	127.5 ± 42.9 #△	**< 0.001**
Lipid volume index, °	2299.8 ± 1237.7	2154.2 ± 1279.2	1277.4 ± 888.3 #△	**< 0.001**
Lipid length, mm	13.8 ± 6.2	13.5 ± 7.0	9.7 ± 5.8 #△	**< 0.001**
Presence of macrophages, n (%)	140 (95.2)	82 (97.6)	251 (92.6)	0.188
Mean macrophage arc, °	55.8 ± 20.0	57.0 ± 19.6	49.7 ± 23.7 #△	**0.004**
Macrophage volume index, °	589.3 ± 448.9	561.4 ± 415.8	389.1 ± 309.9 #△	**< 0.001**
Macrophage length, mm	9.7 ± 6.0	9.4 ± 6.2	7.1 ± 4.7 #△	**< 0.001**
Microvessel, n (%)	52 (35.4)	36 (42.9)	133 (49.1) △	**0.026**
Cholesterol crystal, n (%)	43 (29.3)	17 (20.2)	85 (31.4) #	0.144
Calcium node, n (%)	0 (0)	3 (3.6) *	6 (2.2)	0.059
Mean calcium arc, °	45.1 ± 36.0	56.0 ± 45.3	56.9 ± 47.0△	**0.026**
Calcium length, mm	4.4 ± 6.1	5.5 ± 6.1	6.4 ± 7.6 △	**0.021**
Thrombus, n (%)	124 (84.4)	39 (46.4) *	48 (17.7) #△	**< 0.001**
Plaque rupture, n (%)	96 (65.3)	30 (35.7) *	43 (15.9) #△	**< 0.001**

∗Significance between FCT ≤ 65 μm group and 65 < FCT ≤ 80 μm group; #significance between 65 < FCT ≤ 80 μm group and FCT>80 μm group; △significance between FCT ≤ 65 μm group and FCT>80 μm group. Values are n(%) or mean ± SD

### Univariate and multivariate logistic analyses for plaque rupture and thrombosis

**Fig. 1:**
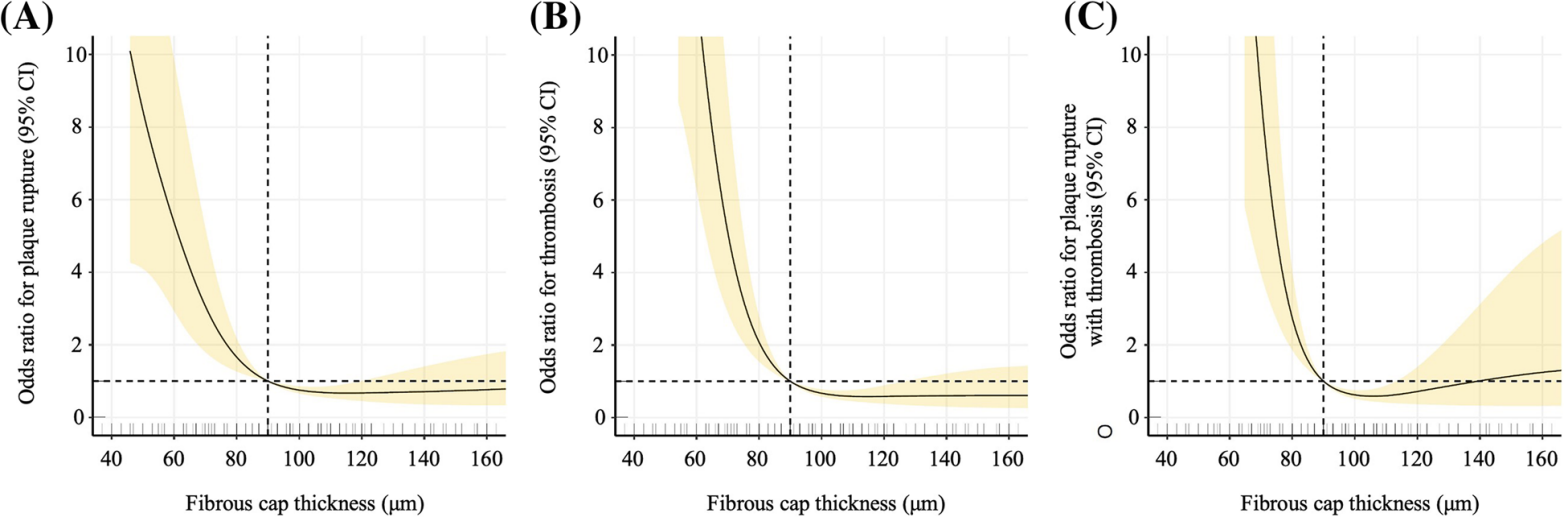
Spline analyses of plaque rupture (**A**), thrombosis (**B**), and plaque rupture with thrombosis (**C**) by the thinnest cap thickness. The models were adjusted for age, sex, diabetes, hypertension, current smoking, LDL-C, crea, hs-CRP and mean lipid arc. Odds ratios are indicated by solid lines and 95% confidence intervals by shaded areas. The reference point is the lowest value for the thinnest cap thickness, with knots placed at the 10th, 30th, 50th, 70th, and 90th centiles of the thinnest cap thickness distribution

The association of FCT and plaque rupture and/or thrombosis (Table 4) was analysed by univariate and multivariate logistic analyses. FCT ≤ 65 μm and 65 < FCT ≤ 80 μm were independent predictors for plaque rupture ([FCT ≤ 65 μm vs. FCT > 80 μm]: OR = 8.082, 95% CI = 4.861 to 13.435, *P* < 0.001; [65 < FCT ≤ 80 μm vs. FCT > 80 μm]: OR = 2.463, 95% CI = 1.370 to 4.430, *P* = 0.003) (Table 4A), thrombosis ([FCT ≤ 65 μm vs. FCT > 80 μm]: OR = 25.224, 95% CI = 13.768 to 46.212, *P* < 0.001; [65 < FCT ≤ 80 μm vs. FCT > 80 μm]: OR = 3.675, 95% CI = 2.065 to 6.542, *P* < 0.001) (Table 4B) and plaque rupture with thrombosis ([FCT ≤ 65 μm vs. FCT > 80 μm]: OR = 22.593, 95% CI = 11.426 to 44.674, *P* < 0.001; [65 < FCT ≤ 80 μm vs. FCT > 80 μm]: OR a= 4.143, 95% CI = 1.869 to 9.184, *P* < 0.001) (Table 4C). In Fig. 1, restricted cubic splines were used to flexibly model and visualize the relation between FCT and plaque rupture and/or thrombosis. The risk of plaque rupture and thrombosis gradually decreased until FCT reaches approximately 80 μm and then remained relatively flat afterwards (*P* for nonlinearity < 0.001).

**Table 4:** Univariable and multivariable logistic analyses for plaque rupture and thrombus

(A) Plague rupture							
Cap thickness (μm)	Cases/Overall (%)	Model 1		Model 2		Model3	
		Odds ratio [95% CI]	*P*-value	Odds ratio [95% CI]	*P*-value	Odds ratio [95% CI]	*P*-value
FCT ≤ 65	96/147 (65.3)	9.981 [6.234 to 15.980]	**< 0.001**	9.778 [5.985 to 15.977]	**< 0.001**	8.082 [4.861 to 13.435]	**< 0.001**
65 < FCT ≤ 80	30/84 (35.7)	2.946 [1.695 to 5.119]	**< 0.001**	2.911 [1.645 to 5.151]	**< 0.001**	2.463 [1.370 to 4.430]	**0.003**
FCT > 80	43/271 (15.9)	1 (Reference)		1 (Reference)		1 (Reference)	
*P* for trend			**< 0.001**		**< 0.001**		**< 0.001**
(B) Thrombus							
Cap thickness (μm)	Cases/Overall (%)	Model 1		Model 2		Model3	
		Odds ratio [95% CI]	*P*-value	Odds ratio [95% CI]	*P*-value	Odds ratio [95% CI]	*P*-value
FCT ≤ 65	124/147 (84.4)	25.047 [14.547 to 43.126]	**< 0.001**	28.756 [15.949 to 51.847]	**< 0.001**	25.224 [13.768 to 46.212]	**< 0.001**
65 < FCT ≤ 80	39/84 (46.4)	4.026 [2.369 to 6.842]	**< 0.001**	4.119 [2.352 to 7.213]	**< 0.001**	3.675 [2.065 to 6.542]	**< 0.001**
FCT > 80	48/271 (17.7)	1 (Reference)		1 (Reference)		1 (Reference)	
*P* for trend			**< 0.001**		**< 0.001**		**< 0.001**
(C) Plague rupture with thrombosis							
Cap thickness (μm)	Cases/Overall (%)	Model 1		Model 2		Model3	
		Odds ratio [95% CI]	*P*-value	Odds ratio [95% CI]	*P*-value	Odds ratio [95% CI]	*P*-value
FCT ≤ 65	87/147 (59.2)	26.618 [14.170 to 50.002]	**< 0.001**	26.842 [13.818 to 51.144]	**< 0.001**	22.593 [11.426 to 44.674]	**< 0.001**
65 < FCT ≤ 80	18/84 (21.4)	5.006 [2.367 to 10.588]	**< 0.001**	4.825 [2.212 to 10.523]	**< 0.001**	4.143 [1.869 to 9.184]	**< 0.001**
FCT > 80	14/271 (5.2)	1 (Reference)		1 (Reference)		1 (Reference)	
*P* for trend			**< 0.001**		**< 0.001**		**< 0.001**

Model 1 unadjusted

Model 2 adjusted for age, sex, smoking status, diabetes, hypertension, LDL-C, Creatinine, and hs-CRP

Model 3 adjusted for age, sex, smoking status, diabetes, hypertension, LDL-C, Creatinine, hs-CRP, and mean lipid arc

## Discussion

In the present study, plaques with a 65 < FCT ≤ 80 μm were more probable to rupture and/or thrombosis than that of FCT > 80 μm. Moreover, FCT ≤ 65 μm and 65 < FCT ≤ 80 μm were independent predictors of plaque rupture and/or thrombosis.

Vulnerable plaque is defined as a plaque with a high risk of rupture and be more likely to cause ACS [6, 7]. However, the identification of high-risk plaque characteristics has not been able to well predict the occurrence of cardiovascular risk events [8]. Even some vulnerable plaques did not rupture and were asymptomatic clinically [6, 9]. This has led to the concept of vulnerable plaques being challenged recently. Moreover, although some plaques are not vulnerable, they also have a high risk of rupture and thrombosis, leading to ACS events [9, 10]. Plaque rupture usually originates from an excessive stress concentration on the fibrous cap, which trespasses the tensile strength of the material and ultimately causes material failure and consequently plaque rupture. The fibrous cap provides, in this view, a protective effect, by avoiding the exposure of prothrombotic material contained in the necrotic lipid core. Thus, minimal FCT has been considered the most established indicator of plaque vulnerability.

The pathological features of vulnerable plaques include thin fibrous caps, large necrotic cores, macrophage infiltration within the plaque, positive remodelling, cholesterol clefts and neovascularization [6, 7, 11]. Current view of point is that thickening of the fibrous cap can prevents a lipid-containing atheromatous plaque from rupturing. Therefore, the thickness of the fibrous cap is the key factor to determine plaque vulnerability. Pathology studies have defined a FCT < 65 μm as the cut-off threshold of vulnerable plaques. This cut-off value was derived from morphometric analysis of 41 ruptured plaques, of which 95% had a FCT less than 64 μm [6, 11]. However, although plaques with thin fibrous cap< 65 μm are generally considered vulnerable plaques, tissue shrinkage from tissue biopsy may affect morphological measurements [2], and some reports have used higher thresholds (> 200 μm) to define vulnerable plaques [12, 13].

OCT, with its high axial resolution, is the only in vivo modality that can be used to measure the FCT of an atheromatous plaque [14, 15]. The sensitivity, specificity and overall diagnostic accuracy were 92, 99 and 99%, respectively [16]. Tian et al. [17] showed that OCT was the best method to differentiate a ruptured plaque from a non-ruptured TCFA. Yonetsu et al. [2] evaluated 71 ruptured plaques by OCT and found that 95% of ruptured plaques had a FCT < 80 μm. Kubo et al. [18] demonstrated with OCT measurements that the median of thinnest FCT was 54 μm (range 50–60 μm) and 80 μm (67–104 μm) in 71 ruptured plaques and 111 non-ruptured plaques, respectively. Prati et al. reported that the simultaneous presence of multiple OCT high-risk coronary plaque features including FCT < 75 mm, lipid arc extension > 180° in patients with major coronary events in the first year of follow-up in the CLIMA study [19]. Research mentioned above shows that the current risk predicting model of plaque rupture needs to be improved.

In order to improve the predictive value, better detection technology of vulnerable plaques is needed. The CLIMA study explored the correlation between the coronary plaque morphology and the risk of major cardiovascular event by using OCT to detect high-risk plaques. The simultaneous presence of MLA < 3.5 mm2, FCT < 75 mm, lipid arc extension > 180°, and OCT-defined macrophage infiltration in the same coronary plaque was confirmed as an independent predictor of events [19]. Otherwise, the relationship between plaque rupture and the balance of stabilizing and destabilizing forces in the plaque remains unexplored. Whether plaque rupture occurs at the site with the smallest FCT, which represents the point of the least resistance, or rather in sites of highest stress concentration, indicates the point of maximal disruption, is still unclear. Milzi et al. generated patient-specific reconstructions of coronary plaques, which were used to analyse stress concentration as a possible predictor of plaque rupture and is a feasible tool to determine plaque biomechanics [20].

The presence of a large lipid pool, thin fibrous cap, and marked inflammatory cell infiltration are essential characteristics of plaques prone to rupture then trigger potentially fatal coronary events [21]. Intensive statin therapy has been shown to halt the progression of coronary atheroma burden [22] and might affect plaque composition by reducing plaque lipid content and increasing the thickness of the fibrous cap [23]. Raber et al. showed significantly PAV regression, massive lipid burden reduction and greater increase of minimal FCT in patients treated with intensive statin therapy [23]. Taken together, intensive lipid-lowering therapy is an important method to intervene vulnerable plaques.

In the current study, it is identified by ROC analysis that the cut-off value of cap thickness used for predicting plaque rupture and/or thrombosis was approximately 80 μm (81.5 μm, 78.5 μm, and 74.5 μm for plaque rupture, thrombosis, and plaque rupture with thrombosis, respectively) (Supplement Fig. 1). Plaques with 65 < FCT ≤ 80 μm had a higher probability of rupture and thrombosis than that of FCT > 80 μm. Future prospective in vivo studies are warranted to validate whether FCT ≤ 80 μm could predict plaque rupture or clinical events or serve as a therapeutic target for patients with CAD.

### Limitations

Several limitations of this study should be acknowledged. First, this study employed a retrospective design with selection bias, and the findings must be confirmed in a prospective study. Second, the sample size of this study was relatively small, although we needed a long period to collect the data. Finally, plaque vulnerability was determined only by OCT; the combined use of OCT and intravascular ultrasound (IVUS) may improve plaque burden detection accuracy.

## Conclusion

Plaques with a 65 < FCT ≤ 80 μm was an independent predictor for plaque rupture and/or thrombosis.

## Supplementary Information

**Additional file 1: Supplement Figure 1.** Receiver operating characteristic curves for measurements of fibrous cap thickness for prediction of plaque rupture (A), thrombosis (B) or plaque rupture with thrombosis (C). AUC, area under the curve.

## Data Availability

All data generated or analysed during this study are included in this published article [and its supplementary information files].
