# Supplementary material for: New insights into fibrous cap thickness of vulnerable plaques assessed by optical coherence tomography
Source: BMC Cardiovasc Disord. 2022 Nov 12;22:484. doi: 10.1186/s12872-022-02896-z (PMC9655862; doi:10.1186/s12872-022-02896-z)

**Supplement figure captions**

**Supplement Figure 1.** Receiver operating characteristic curves for measurements of fibrous cap thickness for prediction of plaque rupture (A), thrombosis (B) or plaque rupture with thrombosis (C). AUC, area under the curve.


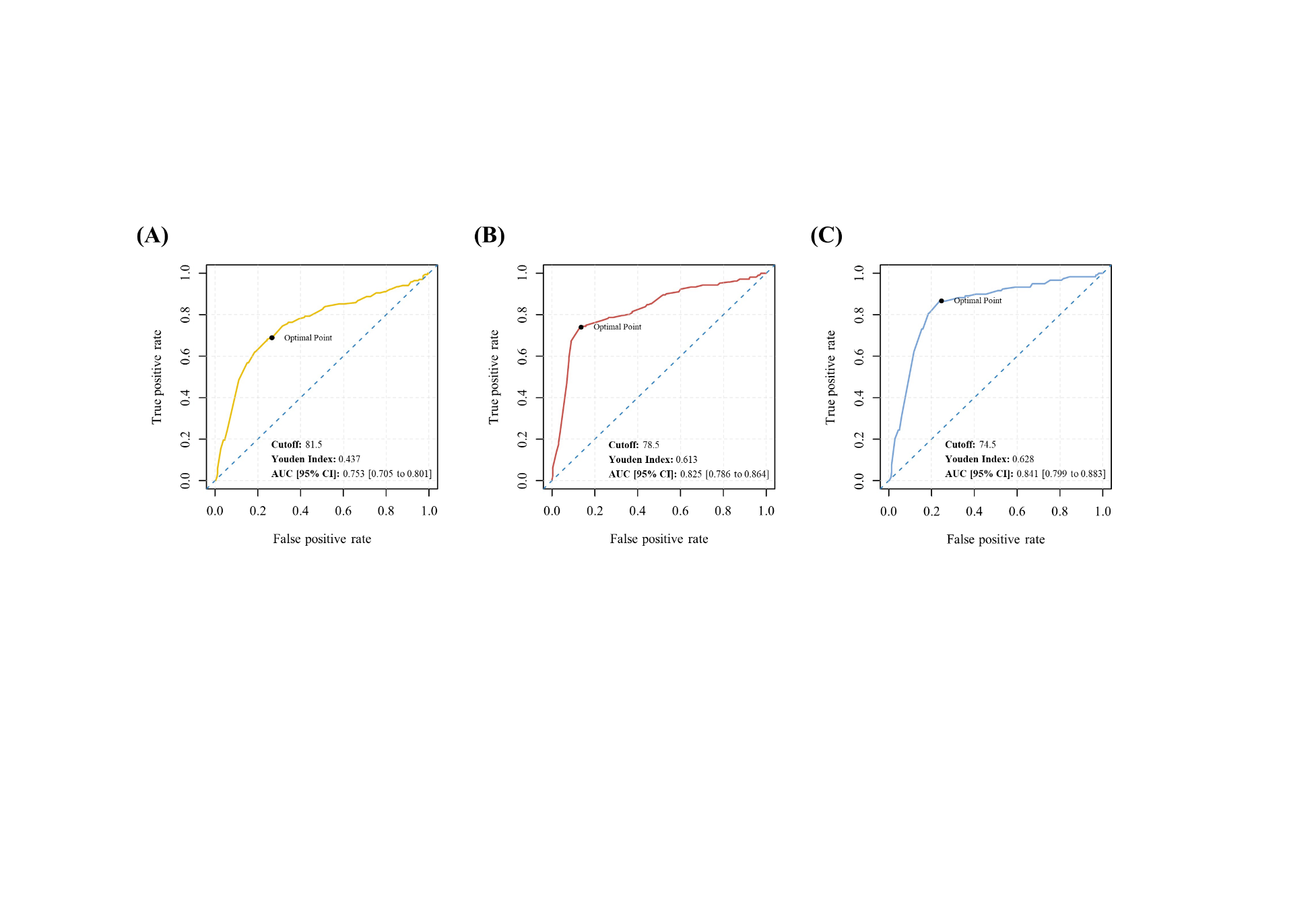

Supplement: Supplementary file 1 — Additional file 1: Supplement Figure 1. Receiver operating characteristic curves for measurements of fibrous cap thickness for prediction of plaque rupture (A), thrombosis (B) or plaque rupture with thrombosis (C). AUC, area under the curve. [file 12872_2022_2896_MOESM1_ESM.docx]
